# Hypothalamic sensing of ketone bodies after prolonged cerebral exposure leads to metabolic control dysregulation

**DOI:** 10.1038/srep34909

**Published:** 2016-10-06

**Authors:** Lionel Carneiro, Sarah Geller, Audrey Hébert, Cendrine Repond, Xavier Fioramonti, Corinne Leloup, Luc Pellerin

**Affiliations:** 1 Department of Physiology, University of Lausanne, 1005 Lausanne, Switzerland; 2 UMR CNRS 6265-INRA 1324-Univ. Bourgogne Franche-Comté Centre des sciences du goût et de l’alimentation, 21000 Dijon, France

## Abstract

Ketone bodies have been shown to transiently stimulate food intake and modify energy homeostasis regulatory systems following cerebral infusion for a moderate period of time (<6 hours). As ketone bodies are usually enhanced during episodes of fasting, this effect might correspond to a physiological regulation. In contrast, ketone bodies levels remain elevated for prolonged periods during obesity, and thus could play an important role in the development of this pathology. In order to understand this transition, ketone bodies were infused through a catheter inserted in the carotid to directly stimulate the brain for a period of 24 hours. Food ingested and blood circulating parameters involved in metabolic control as well as glucose homeostasis were determined. Results show that ketone bodies infusion for 24 hours increased food intake associated with a stimulation of hypothalamic orexigenic neuropeptides. Moreover, insulinemia was increased and caused a decrease in glucose production despite an increased resistance to insulin. The present study confirms that ketone bodies reaching the brain stimulates food intake. Moreover, we provide evidence that a prolonged hyperketonemia leads to a dysregulation of energy homeostasis control mechanisms. Finally, this study shows that brain exposure to ketone bodies alters insulin signaling and consequently glucose homeostasis.

Energy homeostasis can be maintained by the activation of several mechanisms involving an interplay between the central nervous system (CNS) and peripheral organs. Among the different parameters regulated to maintain energy supply and expenditure on balance, body weight and glycemia are normally kept constant. This balance is due to different circulating or nervous signals allowing peripheral organs to communicate between them and with the CNS^1^,^2^. The CNS plays an important role by integrating different endocrine, nervous and nutrient signals^3^. After information treatment, it can send to peripheral organs a regulatory signal via the Autonomic Nervous System (ANS). Thus, the CNS can modulate liver, adipose tissue, pancreas or muscle activity but also directly adapt food intake and energy expenditures, to maintain body weight and internal parameters such as glycemia.

Such brain control of energy homeostasis was shown to be altered during obesity or metabolic disorders^4^. Thus, recently, numerous studies have attempted to unravel the role of nutrient sensing of various brain areas and their involvement in energy homeostasis regulation^5^. Of interest is the hypothalamus due to its particular location in the most ventral part of the brain, near the 3^rd^ ventricle and the polygon of Willis, which makes it one of the first brain area reached by circulating nutrients. In addition, at this level the blood-brain barrier is more fenestrated and the concentrations of nutrients in the brain parenchyma at this location are close to the ones measured in the blood^6^,^7^,^8^,^9^,^10^. Glucose is the main source of energy for the brain and is also a key signal for the hypothalamic control of metabolism^11^,^12^. However, during specific conditions, it has been shown that the central nervous sytem can also rely on other energy sources such as fatty acids or ketone bodies^13^,^14^,^15^. However, among the different signals involved in such a regulatory loop, ketone bodies remain poorly studied. Indeed, the role of ketone bodies as energetic signal was already suggested decades ago^16^,^17^,^18^. The ability of the brain to use this substrate as an alternative energetic supply has been highlighted notably by the study of Hawkins *et al*.^13^. Under basal conditions, brain utilization of ketone bodies is limited because their blood concentration is low (<0.3 mM). However, during fasting periods, obesity, or type 1 diabetes, their concentration increases and the brain starts to significantly use ketone bodies when their concentration rises above 4 mM, which corresponds to the K_m_ of the ketone bodies transporter MCT1 expressed by the cerebral blood vessels^13^,^19^,^20^,^21^. Interestingly, the hypothalamus exhibits a higher ability to metabolize ketone bodies than other brain regions^22^. Nevertheless, only recent studies have unraveled an impact of ketone bodies on brain control of energy homeostasis^23^,^24^,^25^,^26^,^27^,^28^.

It was recently demonstrated that a short-term infusion (<6 hours) of ketone bodies to the brain through an intracarotidic route led to a subsequent deregulation of energy homeostasis^29^. An increased food intake was evidenced associated with an overexpression of orexigenic neuropeptides in the hypothalamus together with an activation of the AMPK (known to be activated before food intake is stimulated). In addition, a perturbation of glucose homeostasis was observed due to increased insulinemia. In accordance, the hepatic glucose production was decreased. Finally, this phenotype was only observed after 6 hours of infusion. When the infusion lasted 12 hours, all these parameters were restored to their normal level. This previous work indicated that hyperketonemia can be detected by the CNS as a signal of energy deficit that triggers a counterregulatory response leading to a normalization of metablic parameters after 12 hours^29^. These previous results are in agreement with data reviewed recently by Paoli *et al*. and could help to explain contradictory observations concerning orexigenic vs. anorexigenic effects of ketone bodies^30^. Most of the previous studies were performed using a ketogenic diet and showed a decrease in food intake, due at least in part to astrocytic ketogenesis and fatty acids^23^,^31^,^32^,^33^,^34^,^35^,^36^. More recently, a short-term exposure to a high fat diet inducing an increase in circulating fatty acids and ketonemia was used to study the role of ketone bodies in the central control of energy homeostasis. The results presented by the authors support the idea that astrocytic ketogenesis in the hypothalamus induced by an increase in fatty acid metabolism is responsible for the food intake inhibition observed after 3 days of high fat diet^36^,^37^. Such results are contradictory with our previous results^29^, but the different strategies used (high fat diet vs carotidic infusion) associated with different time points (3 days vs 12 h) and in different animal models (rats vs mice) most likely explain such differences. The reversal of the effect observed in our model with time suggests that a biphasic effect could occur and lead to the opposite effect at later time points, which would reconciliate both views. However, it is important to emphasize that as ketone bodies are produced under normal conditions during fasting periods, hyperketonemia is transient and occurs only during a short-time period. In this regard, a short-term infusion of 12 hours mimics a physiological increase in ketonemia and it activates the normal adaptive mechanism during a fast. Notwithstanding, these previous studies concur to indicate that a modification of ketone bodies levels, whether on a short-term or a long-term basis, plays a particular and complex role in the control of energy homeostasis that is still far from being understood.

To further understand how ketone bodies sensed by the central nervous system modify energy homeostasis, we decided to investigate the effect of a prolonged (>12 hours) ketone bodies infusion to the brain. Our hypothesis is that the physiological counterregulatory response previously unraveled after a 12-hour period might recede over a longer exposure time period, paving the way to pathophysiological alterations later on.

## Results

### Food intake and blood profile modifications induced by a 24h infusion of β-hydroxybutyrate (BHB) to the brain

**Figure 1:**
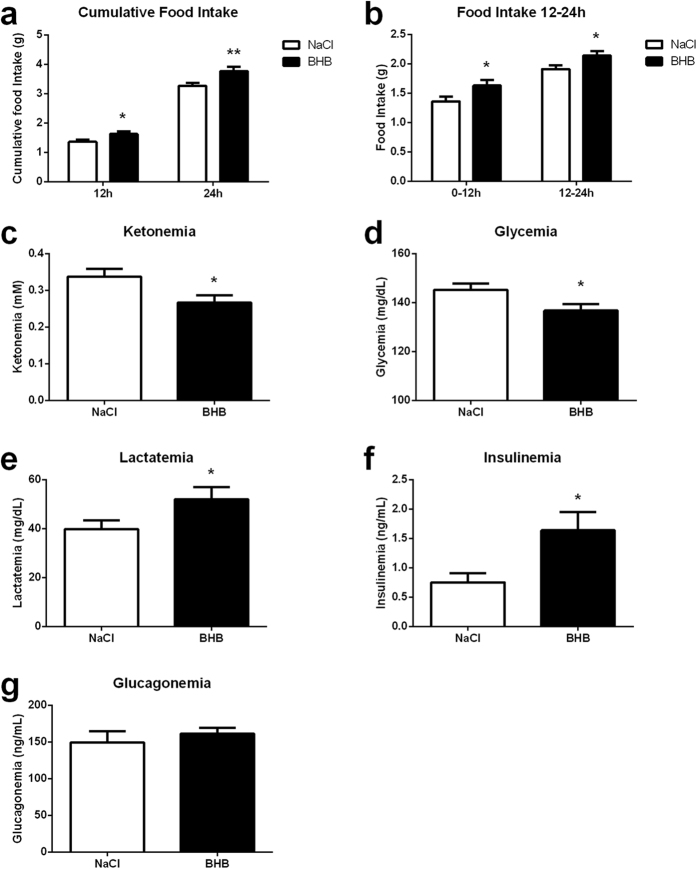
Effect of intracarotid BHB infusion on food intake as well as on blood parameters. (**a**) Cumulative food intake determined at 12 and 24 h of NaCl or BHB infusion by subtracting the weight of food pellet remaining from the weight of food provided at the start of the experiment. (**b**) Food ingested determined between 0 and 12 h and between 12 and 24 h of NaCl or BHB infusion by subtracting the weight of food pellet remaining from the weight of food provided at the previous time of measurement of the experiment. (**c**) Ketonemia at 24 h in mice infused with either NaCl or BHB (**d**) Glycemia at 24 h in mice infused with either NaCl or BHB (**e**) Lactatemia at 24 h in mice infused with either NaCl or BHB (**f**) Insulinemia at 24 h in mice infused with either NaCl or BHB (**g**) Glucagonemia at 24 h in mice infused with either NaCl or BHB. Data represent the mean ± SEM with n = 8–24 animals per condition and were statistically analyzed with an unpaired Student t-test. *p < 0.05 vs. NaCl; **p < 0.01 vs. NaCl. a.u. arbitrary units.

Analysis of the cumulated food intake in mice revealed a significant increase after both 12 hours and 24 hours of β-hydroxybutyrate infusion via an intracarotidic route compared to those infused with NaCl (Fig. 1a). When the analysis was performed specifically on the first or the last 12 hour period of BHB infusion (0–12 h and 12–24 h, respectively), measurements revealed a significant enhancement of food intake for both periods (Fig. 1b). Then, the blood concentrations of the main energy substrates involved in energy homeostasis regulation were determined in BHB and NaCl infused mice. Ketonemia measured at 24 hours was significantly decreased in the BHB group vs. the NaCl group (Fig. 1c). Glycemia was also significantly reduced in BHB infused mice compared to NaCl infused mice (Fig. 1d). In contrast, lactatemia was significantly increased in the BHB group compared to the NaCl group (Fig. 1e). Finally, blood concentrations of insulin and glucagon participating to the regulation of carbohydrate homeostasis were measured in BHB and NaCl infused mice. Insulinemia increased following 24 hours of BHB infusion compared to the animals infused with NaCl (Fig. 1f). Glucagon levels on the other hand were not different between the two groups (Fig. 1g).

### Expression of genes involved in food intake regulation and ketone bodies metabolism upon BHB infusion

**Figure 2:**
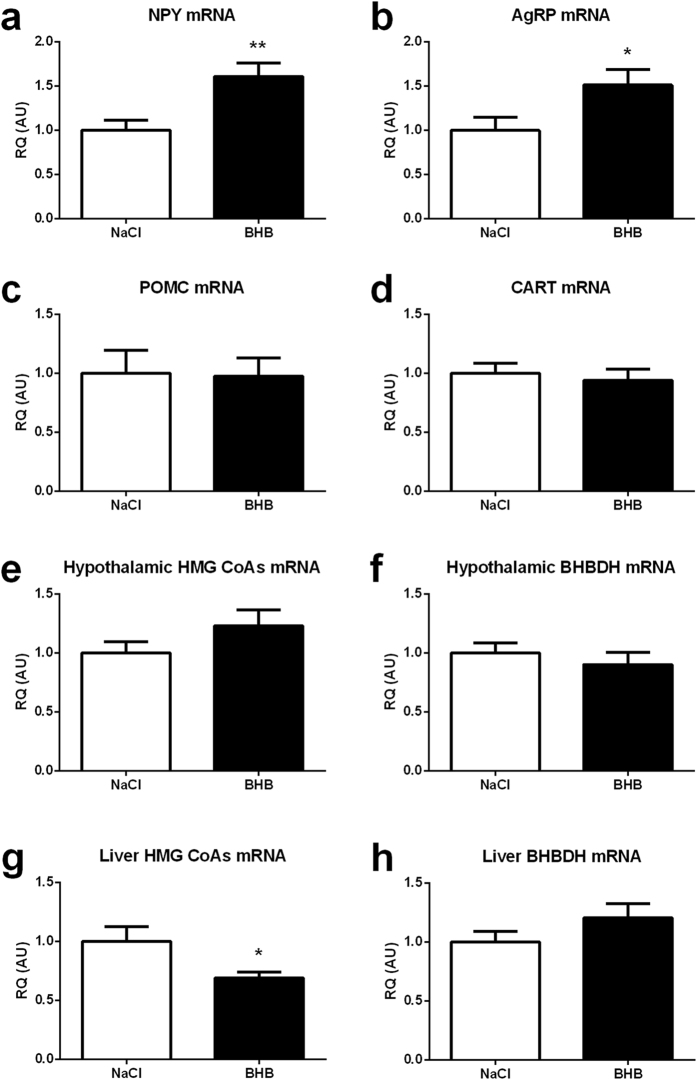
Effect of intracarotid BHB infusion on orexigenic and anorexigenic hypothalamic neuropeptide mRNA expression as well as on the mRNA expression of ketogenic enzymes in the hypothalamus and in the liver. (**a**) Hypothalamic NPY mRNA expression at 24 h in mice infused with either NaCl or BHB (**b**) Hypothalamic AgRP mRNA expression at 24 h in mice infused with either NaCl or BHB (**c**) Hypothalamic POMC mRNA expression at 24 h in mice infused with either NaCl or BHB (**d**) Hypothalamic CART mRNA expression at 24 h in mice infused with either NaCl or BHB. (**e**) Hypothalamic HMG-CoAs mRNA expression at 24 h in mice infused with either NaCl or BHB. (**f**) Hypothalamic BHBDH mRNA expression at 24 h in mice infused with either NaCl or BHB. (**g**) Liver HMG-CoAs mRNA expression at 24 h in mice infused with either NaCl or BHB. (**h**) Liver BHBDH mRNA expression at 24 h in mice infused with either NaCl or BHB. mRNA expression for each hypothalamic peptide and enzyme involved in ketone bodies metabolism was determined by quantitative reverse transcriptase–PCR at 24 h of NaCl or BHB infusion. Data represent the mean ± SEM with n = 9–12 animals per condition and were statistically analyzed with unpaired Student t-test. *p < 0.05 vs. NaCl; **p < 0.01 vs. NaCl. a.u., arbitrary units.

Determination of the mRNA expression levels of the main hypothalamic neuropeptides regulating food intake revealed an increased expression of the orexigenic neuropeptides NPY and AgRP (Fig. 2a,b) following 24 hours of cerebral BHB infusion. mRNA expression levels of the anorexigenic neuropeptides POMC and CART were not significantly modified following 24 hours of cerebral BHB infusion (Fig. 2c,d, respectively).

It was important to determine whether the capacity to metabolize ketone bodies was modified in the hypothalamus following the intracarotid infusion of BHB. For this purpose, the expression levels of the mRNAs coding for 3-Hydroxy-3-methylglutaryl-CoA synthase (HMG CoA synthase), the enzyme responsible of ketone bodies production, and for 3-Hydroxybutyrate dehydrogenase (BHBDH), the enzyme involved in the utilization of ketone bodies, were determined after either NaCl or BHB infusion. In parallel, a similar investigation was performed in the liver, a tissue which was not directly exposed to BHB with our experimental approach. In the hypothalamus, the expression of the HMG CoA synthase mRNA and the BHBDH mRNA is similar between NaCl infused and BHB infused mice (Fig. 2e,f). However, in the liver, if the expression of the BHBDH mRNA remained unchanged (Fig. 2g), the HMG CoA synthase mRNA level decreased significantly in BHB-infused mice compared to NaCl-infused mice (Fig. 2h).

### Putative hypothalamic AMPK activation as well as hypothalamic and cortical monocarboxylate transporter protein expression following BHB infusion

**Figure 3:**
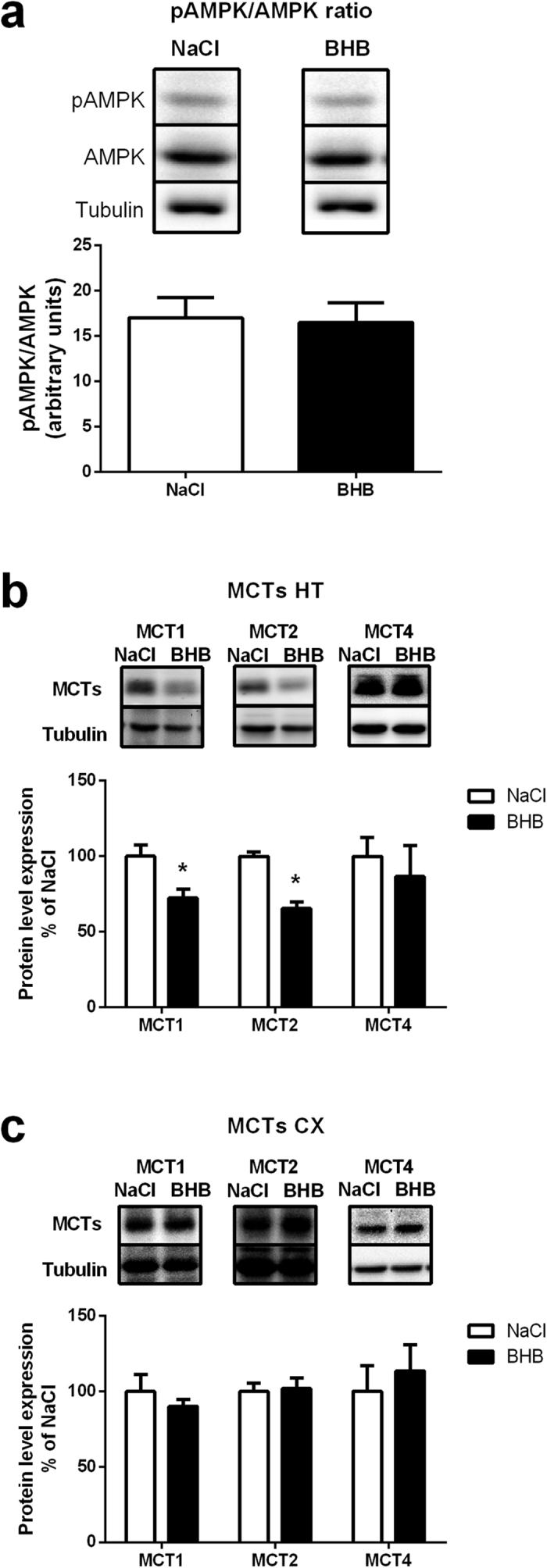
Hypothalamic pAMPK/AMPK ratio, as well as MCT1, MCT2 and MCT4 protein expression in the hypothalamus and cortex following intracarotid BHB infusion. (**a**) Hypothalamic pAMPK/AMPK ratio at 24 h in mice infused with either NaCl or BHB. Upper panels are representative Western blots. Lower panel provides the quantification of protein expression and the resulting ratio (**b**) Hypothalamic MCT1, MCT2 and MCT4 protein expression at 24 h in mice infused with either NaCl or BHB. Upper panels are representative Western blots. Lower panel provides the quantification of protein expression (**c**) Cortical MCT1, MCT2 and MCT4 protein expression at 24 h in mice infused with either NaCl or BHB. Upper panels are representative Western blots. Lower panel provides the quantification of protein expression. MCT protein levels in BHB groups are expressed as percentage of the expression in the corresponding NaCl treated group (set at 100%). β-tubulin was used as internal reference. Data represent the mean ± SEM with n = 7 animals per condition and were statistically analyzed with unpaired Student t-test. *p < 0.05 vs. NaCl.

AMPK is an important intracellular sensor of the energetic state. The hypothalamic expression levels of both AMPK and pAMPK were determined after 24 hours of BHB or NaCl infusion. No alteration in the hypothalamic pAMPK/AMPK ratio was detected after 24 hours of BHB infusion compared to NaCl infusion (Fig. 3a). Considering that monocarboxylate transporters (MCTs) might constitute a limiting step for the use (and eventually detection) of ketone bodies by brain cells, the level of protein expression of MCT1, MCT2 and MCT4 was assessed in both the hypothalamus and the cortex of mice perfused with either NaCl or BHB for 24 hours. Results show that both hypothalamic MCT1 and MCT2 expressions are decreased significantly in BHB-infused mice compared to NaCl-infused mice. The astrocyte-specific isoform MCT4 remained unaffected by the intracarotid BHB infusion (Fig. 3b). A similar determination in the cortex did not reveal any modification (Fig. 3c).

### Peripheral gluconeogenesis during intracarotid BHB infusion

**Figure 4:**
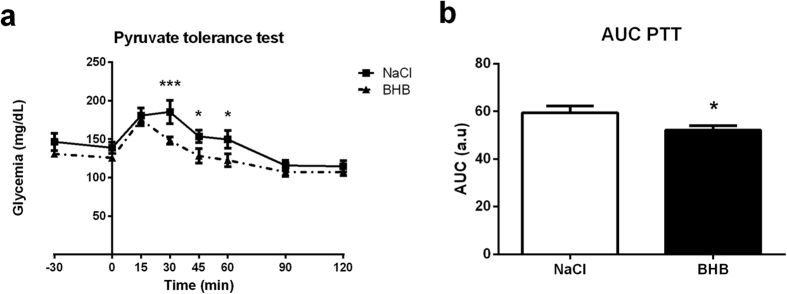
Effect of intracarotid BHB infusion on gluconeogenesis. (**a**) Intraperitoneal pyruvate tolerance test performed at 24 h of BHB infusion. (**b**) Area under curve of plasma glucose levels during the Pyruvate tolerance test. Glycemia was measured during the 2 h following an intraperitoneal load of pyruvate which stimulates endogenous glucose production. Data represent the mean ± SEM with n = 6 animals per condition and were statistically analyzed with a two-way ANOVA for (**a**) and an unpaired Student t-test for (**b**). *p < 0.05 vs. NaCl; **p < 0.01 vs. NaCl; ***p < 0.001 vs. NaCl. a.u., arbitrary units; AUC, area under the curve.

**Figure 5:**
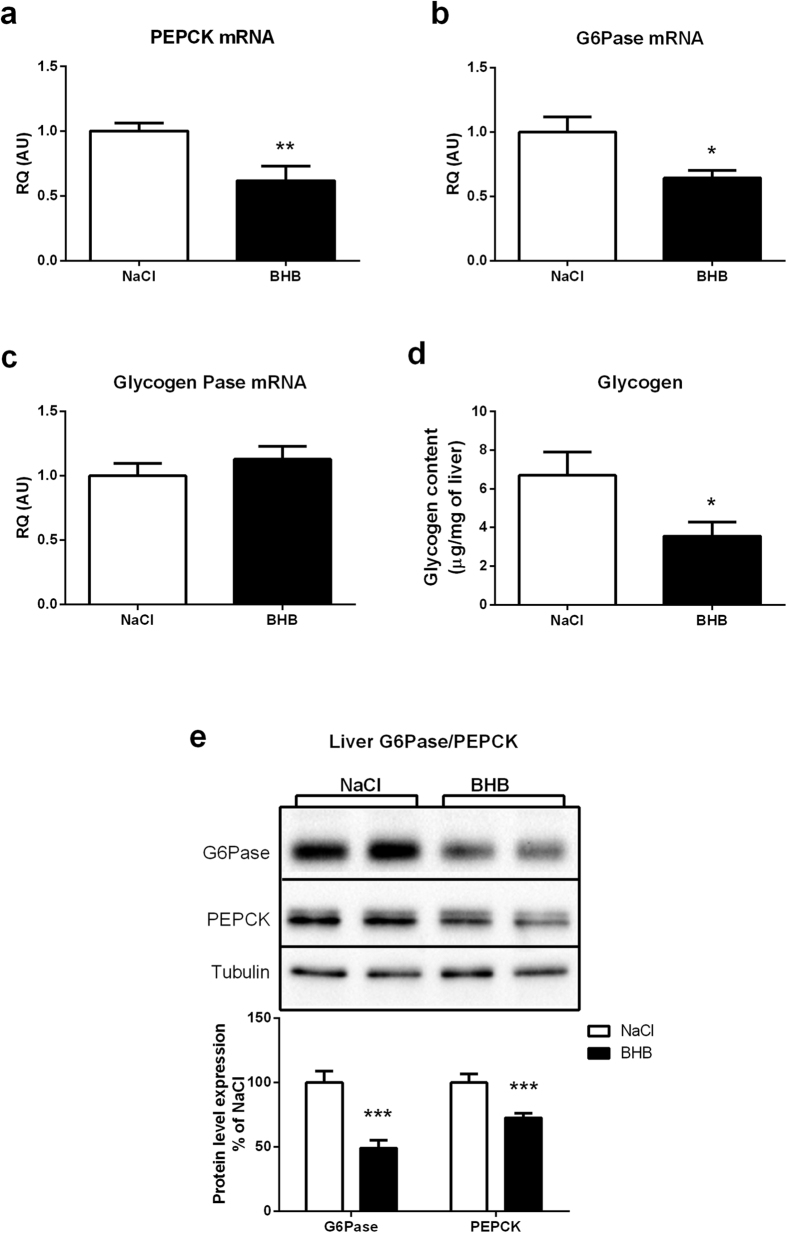
Effect of intracarotid BHB infusion on mRNA expression of liver enzymes involved in gluconeogenesis, glucose production and glycogen synthesis as well as on liver glycogen content. (**a**) PEPCK mRNA expression at 24 h in mice infused with either NaCl or BHB (**b**) G6Pase mRNA expression at 24 h in mice infused with either NaCl or BHB (**c**) GlycogenPase mRNA expression at 24 h in mice infused with either NaCl or BHB (**d**) Liver glycogen content at 24 h in mice infused with either NaCl or BHB. mRNA expression for each enzyme was determined by quantitative reverse transcriptase–PCR at 24 h of NaCl or BHB infusion. Glycogen content was determined by the quantification of the glucose amount produced from glycogen hydrolysis. (**e**) Liver G6Pase and PEPCK protein expression at 24 h in mice infused with either NaCl or BHB. Upper panels are representative Western blots. Lower panel provides the quantification of protein expression. G6PAse or PEPCK protein levels in BHB groups are expressed as percentage of the expression in the corresponding NaCl-treated group (set at 100%). β-tubulin was used as internal reference. Data represent the mean ± SEM with n = 12 animals per condition and were statistically analyzed with unpaired Student t-test. *p < 0.05 vs. NaCl; **p < 0.01 vs. NaCl. a.u., arbitrary units.

The increased lactatemia observed after 24 hours of BHB infusion (Fig. 1d) could be due to decreased liver utilization of lactate for glucose production (i.e. reduced gluconeogenesis). This is in accordance with increased insulin levels observed following cerebral BHB infusion (Fig. 1d,f), insulin being a known inhibitor of gluconeogenesis, and a factor reducing glycemia. To test whether gluconeogenesis might be also affected, a pyruvate tolerance test was performed after 24 hours of BHB infusion. Glucose production in response to the pyruvate injection was found to be reduced in mice infused with BHB for 24 hours (Fig. 4a,b). Consistent with these results, the expression level of the main gluconeogenic enzyme Phosphoenolpyruvate carboxykinase (PEPCK) was decreased in the liver of BHB-infused mice compared to NaCl-infused mice at 24 hours at both the mRNA and protein levels (Fig. 5a,e). In addition, the hepatic expression of Glucose-6-phosphatase (G6Pase), the enzyme controlling the last step of the synthesis of glucose at the end of gluconeogenesis, was also decreased at both the mRNA and protein levels, an observation also consistent with a reduction of glucose production by the liver after 24 hours of cerebral BHB infusion (Fig. 5b,e). In accordance, the expression level of Glycogen Phosphorylase, a key enzyme of glycogenolysis, was not modified after 24 hours of intracarotid BHB infusion (Fig. 5c). Concomitantly, the hepatic glycogen content was decreased in BHB-infused mice compared to the NaCl group (Fig. 5d).

### Peripheral insulin resistance following prolonged intracarotid BHB infusion

**Figure 6:**
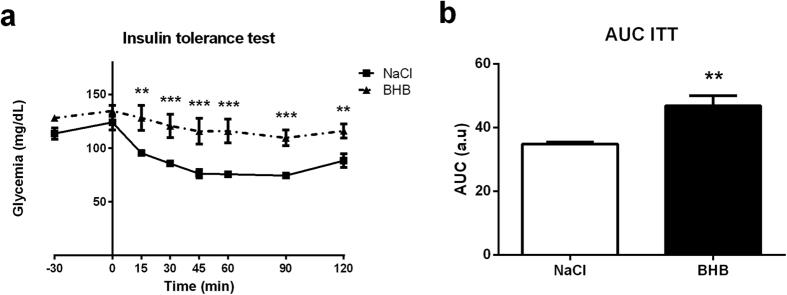
Effect of intracarotid BHB infusion on insulin tolerance. (**a**) Intraperitoneal insulin tolerance test performed at 24 h of BHB infusion. (**b**) Area under curve of plasma glucose levels during the insulin tolerance test. Glycemia was measured during the 2 hours following an intraperitoneal load of insulin which stimulates glucose utilization. Data represent the mean ± SEM with n = 6 animals per condition and were statistically analyzed with a two-way ANOVA for (**a**) and an unpaired Student t-test for (**b**). *p < 0.05 vs. NaCl; **p < 0.01 vs. NaCl; ***p < 0.001 vs. NaCl. a.u., arbitrary units; AUC, area under the curve.

To better assess the significance of the increased insulin levels observed, an insulin tolerance test was done following 24 hours of BHB infusion to determine the sensitivity to insulin. Results revealed an increase of the area under the curve (AUC) in BHB-infused mice after 24 hours of BHB infusion. This observation indicates a decreased sensitivity to the insulin injected intraperitoneally (Fig. 6a,b).

## Discussion

Obesity development is accompanied by alterations in the metabolism of monocarboxylates, such as ketone bodies, or fatty acids (their precursors)^36^,^38^,^39^,^40^. However, it is still not completely understood how monocarboxylates are involved in the physiopathology of obesity and related disorders. Hyperketonemia is observed in high fat diet-induced obesity^15^. In such models, the expression of the ketone bodies transporters (Monocarboxylate transporters or MCTs) is increased in the brain and thus cerebral ketone bodies utilization might increase^15^. In addition, sustained hyperketonemia could be in part responsible for the development of the obese phenotype. Resistance to the high fat diet-induced obesity in the MCT1+/− mouse model confirms the potential role of ketone bodies in obesity development^41^ and supports the notion of monocarboxylates playing an important role in obesity development and associated disorders^40^.

Several studies have indicated that ketone bodies affect the control of energy homeostasis^27^,^28^,^29^,^30^,^36^,^42^,^43^. Of particular interest, it was demonstrated that hypothalamic ketone bodies are responsible for the increased food intake observed in type 1 diabetic rats^27^. Moreover, this study as well as others have reported an increase in the expression level of the anorexigenic neuropeptide AgRP^27^. However, other groups have also described the opposite effect, thus assigning an inhibitory role for ketone bodies on food intake^36^,^44^. Contradictory results could be due to different strategies and/or protocols used^30^. Various approaches to study the role of ketone bodies in food intake control were used including direct delivery of ketone bodies within the brain, subcutaneous injections or ketogenic diets^23^,^24^,^25^,^26^,^28^,^45^. Each of these strategies bypasses the natural supply route (blood circulation) and/or affects other brain regions than the hypothalamus, while some act as well on peripheral organs and thus can not address specifically the role of the hypothalamus alone in ketone bodies sensing. Moreover, ketogenic or high fat diets induce not only increased ketone bodies levels but also led to a rise of other circulating nutrients such as fatty acids. These previous studies strongly support the idea of a key role of ketone bodies sensing (centrally and at peripheral levels) to maintain energy balance. Nevertheless, they did not specifically address the question of the involvement of ketone bodies signaling in food intake regulation due to the presence of other possibly confounding factors (e.g. fatty acids or glycemia).

Recently, we described a transient dysregulation of body energy homeostasis induced by a cerebral ketone bodies infusion^29^. Results of this previous study suggested the activation of regulatory mechanisms by ketone bodies to re-establish body energy homeostasis. In the present study, we wanted to determine the impact of a ketone bodies infusion that would exceed in time what could be considered physiological. Indeed, the duration of 12 hours of infusion used in the first study represents a duration compatible with a physiological response to transient hyperketonemia which occurs during the daily fasting periods^19^,^20^. Thus, we hypothesized that 24 hours of infusion could represent a good model for the transition toward pathological hyperketonemia^46^,^47^. In addition, since 12 hours of ketone bodies infusion is sufficient to overcome the dysregulation observed at 6 hours, we chose to infuse BHB for a time just exceeding this normalizing period to observe whether the counterregulatory response would disappear. In order to address this hypothesis, β-Hydroxybutyrate was infused via the carotid for 24 hours to still stimulate primarily the hypothalamus which is involved in the control of energy homeostasis. As previously described, the infused dose of 20 μg/h is considered to be particularly high compared to the concentration measured during a fast. However, the precise concentration reaching the hypothalamus can not be estimated in a satisfactory manner. Since ketone bodies are infused through the right carotid, the concentration reaching the hypothalamus is expected to be much lower due to the dilution with blood supplied by the second carotid at a higher flow rate (0.75 ml/min blood flow in left carotid vs 30 μl/h infusion rate of BHB in the right carotid). In addition, the infusion is started 5 hours after light extinction to avoid the period of physiological ketogenesis^21^,^22^.

In the same previous study, an increase in food intake at both 6 hours and 12 hours of infusion was described while the other changes induced by ketone bodies injection after 6 hours normalized at 12 hours. Thus, in accordance with results obtained in other studies, an effect of ketone bodies as orexigenic signal could be evidenced^27^,^43^. In the present study, 24 hours of BHB infusion led also to an enhancement of food intake, confirming the orexigenic action of ketone bodies. In the previous short-term exposure study, mice receiving BHB presented an increase in food intake solely between 3 and 6 hours. After this period, BHB infused mice ate a similar quantity of food than the control group until the 12 hours time point. The fact that food intake measured for the 6–12 hour period return to pre-infusion values suggested the possibility of a biphasic effect. Here we report that during the following 12 hours (i.e. between 12 hours and 24 hours of infusion) continuous BHB infusion stimulated again food intake (Fig. 1a,b). Thus, it appears that a prolonged infusion of BHB disrupts the normalized state reached at 12 hours. Such a result suggests that the counterregulation mechanism effective after 12 hours of infusion could be disrupted with a longer infusion period leading to a sustained food intake stimulation. Consistent with this view, the orexigenic neuropeptides NPY and AgRP are also stimulated at the mRNA level (Fig. 2), confirming as previously shown that ketone bodies can be considered as an orexigenic signal^27^,^43^. However, after 24 hours of infusion, this increased food intake is no longer accompanied by a rise in AMPK activation (Fig. 3a). Such a result could be explained by the delayed use of BHB as oxidative substrate after the initial inhibition of glucose uptake and oxidation^43^. BHB oxidation would compensate for the decrease in glucose-based ATP production at least in part to re-establish an appropriate AMP/ATP ratio, thus avoiding the activation of AMPK. Infused ketone bodies could represent an adequate energetic source to maintain ATP production and thus prevent further AMPK phosphorylation. These results are the opposite of previous ones which report a food intake inhibition caused by ketone bodies^36^. The difference could be due to the models used: rats on a high fat diet vs mice with cerebral infusion of ketone bodies. Thus, the changes in fatty acid concentrations in the blood normally observed during a high fat diet (in addition to ketone bodies) could explain the divergent responses. Interestingly, these studies also highlight different roles for astrocytic vs. hepatic ketogenesis. The study of Le Foll *et al*. describes ketogenesis caused by fatty acid oxidation in astrocytes, whereas here, the intracarotidic infusion mostly mimics a peripheral hyperketonemia reaching brain areas through the circulation. Finally, here, the infusion of high levels of ketone bodies certainly exposes the hypothalamus to supraphysiological levels of BHB, but it has the advantage to reveal solely the impact of ketone bodies on the brain without any modification in their peripheral circulating levels.

A decrease in protein expression of the ketone bodies carriers MCT1 and MCT2 found on neurons^29^,^48^ was observed in the hypothalamus, whereas the glial transporter MCT4 was not affected (Fig. 3b,c). Notwithstanding, food intake and the expression of orexigenic neuropeptides were still stimulated despite a possible decrease in ketone bodies supply to hypothalamic neurons due to reduced MCT1 and MCT2 expression. Such results could suggest an increased sensitivity of a particular population of hypothalamic neurons to ketone bodies, allowing to maintain their response to ketone bodies and the subsequent food intake enhancement. The decrease in neuronal MCT expression could represent an adaptive response to the hyperketonemia sensed by these hypothalamic neurons. It is noteworthy that expression of MCT1 was described on some hypothalamic neurons^49^, and in particular on NPY neurons^29^. These results are in accordance with a role of ketone bodies sensing for such orexigenic neurons. Thus, a decreased expression of MCT1 in parallel with a decrease of the specific neuronal isoform MCT2 supports the conclusion of an increased sensitivity to ketone bodies of hypothalamic neurons. Such a decreased expression might represent an adaptive mechanism in response to long-term hyperketonemia caused by the intracarotid infusion of BHB. This observation also supports a deleterious role of increased ketonemia and its implication in obesity development, by dysregulating food intake control. In accordance with this idea, a mouse model deficient for MCT1 displaying a resistance to diet-induced obesity associated with alterations in food intake suggests a key role for monocarboxylates in energy homeostasis regulation^41^. Considering the present results and the localization of MCT1 on NPY neurons, we hypothesized that neurons that are sensitive to circulating ketone bodies are present in the hypothalamic area. Such neurons with a reduced expression of MCT1 could be responsible for the resistance to dysregulated food intake in haploinsufficient MCT1 mice because of a reduction in ketone bodies signaling.

Interestingly, the ketonemia measured in the general blood circulation in mice infused with BHB via the carotid was decreased (Fig. 1c). In addition, a reduction of mRNA levels of HMG CoA synthase in the liver suggests that the activity of the ketogenesis pathway might be reduced (Fig. 2g). Thus a decrease in liver ketone bodies production could explain the decreased ketonemia in animals infused with BHB via the carotid. In accordance, it has been recently shown that decreased ketogenesis occurs during insulin-induced hypoglycemia^50^. In our study, we also observed an increased insulinemia, suggesting an alteration of the brain-to-pancreas axis control. Although our results present some similarities with the mechanisms described in the study of Schiavon *et al*.^50^, it is also possible that the inhibitory effect of insulin on long chain fatty acid transport in mitochondria also explains at least partly the decreased ketogenesis observed as it is known to depend on β-oxidation of fatty acids^51^.

The increased insulinemia observed was associated with a small decrease in glycemia (Fig. 1d,f). However, as hepatic glycogen levels are decreased (Fig. 5d), glycogenolysis could contribute to prevent a more severe decrease in glycemia. In our model, the inhibitory effect of insulin on food intake seems counterbalanced by hypothalamic hyperketonemia detection^52^. A possible explanation for this effect could be the demonstrated inhibitory role of ketone bodies on reactive oxygen species production, which is needed for food intake inhibition induced by insulin acting in the hypothalamus^53^. Moreover, the increased lactatemia observed (Fig. 1e) in parallel with the decreased glycemia could be due to decreased gluconeogenesis highlighted with the pyruvate tolerance test (Fig. 4a,b) which is confirmed by the decreased expression of the main enzymes involved (Fig. 5a–c). As insulin is an inhibitor of hepatic gluconeogenesis, increased insulinemia could be responsible for the reduced glycemia at least in part via a decrease in hepatic glucose production. In addition, the increased lactatemia could be the consequence of a decreased lactate utilization in hepatic gluconeogenesis and/or an increased lactate production from glycogenolysis. As previously discussed, the expression of the common enzyme involved in glucose release at the end of gluconeogenesis and glycogenolysis, G6Pase, is decreased. So, the observed reduction in hepatic glycogen levels might rather serve the purpose of producing lactate rather than providing glucose for release in the blood. Thus, it is likely that glucose provided by glycogen degradation is metabolized via glycolysis and leads to lactate production and release in the blood, causing an increase in lactatemia.

The insulin tolerance test revealed a decrease of insulin sensitivity in BHB-infused mice (Fig. 4c,d). Hyperketonemia has been already demonstrated to be associated with insulin-induced glucose transport inhibition in muscle, suggesting that it decreases insulin sensitivity^42^,^54^. However, in these studies β-hydroxybutyrate is directly in contact with muscles. Although unlikely since ketonemia at 24 hours is not increased but decreased in our study, we can not exclude however that our results could be due to a peripheral diffusion of the centrally infused ketone bodies, even with a transient rise, during the 24 hour period of infusion. The involvement of hypothalamic brain ketone bodies sensing in peripheral insulin sensitivity alteration will need to be addressed further through a more specific and limited stimulation of the appropriate hypothalamic areas.

Altogether, the results presented here confirm the previous study showing that ketone bodies infused to the brain act as an orexigenic signal. Moreover, ketone bodies sensing seems to initiate a counterregulatory response to diminish the hyperketonemia detected. However, experimental conditions maintaining the ketone bodies flux despite this counterregulation lead to a dysregulated body energy homeostasis, notably on glucose homeostasis. Finally, our results strongly suggest that prolonged hyperketonemia could participate to the transition toward the dysregulated energy homeostasis observed in metabolic disorders such as obesity.

## Methods

### Animals

C57BL6 mice (8-weeks-old; Janvier) were individually housed in a controlled environment (12 h light/dark cycle, light on at 7:00 am, 22 °C), with ad libitum access to food and water. Ketone bodies infusion in unrestrained mice was done as described previously in rats and adapted for mice^55^,^56^. Surgery and catheter implantation in the right carotid were performed under pentobarbital anesthesia (50 mg/kg). Experiments were then performed after 1week of recovery. All procedures involving mice followed the European Communities Council Directive (86/609/EEC) and were in accordance with the Swiss animal welfare laws. They were approved by the Service de la consommation et des affaires vétérinaires du Canton de Vaud, Switzerland under the authorization no. VD 2634.

### Feeding test

Unrestrained mice were infused during 24 hours with DL-β-Hydroxybutyric acid (Sigma, Buchs, Switzerland) (50 mM at a rate of 30 μL/h which corresponds to a 20 μg/h of BHB infusion). The infusion started 5 hours after the beginning of the dark period. After 24 hours of infusion, food intake was measured. At the end of the infusion period, mice were sacrificed and hypothalamus, cortex, liver and blood were removed for further analysis.

### Pyruvate and Insulin tolerance tests

Mice received an intraperitoneal pyruvate (2 mg/g) or insulin (0.5 mU/g) injection after 24 hours of ketone bodies infusion. Blood was collected from the tail vein at - 30, 0, 15, 30, 45, 60, 90, and 120 minutes for determination of glucose levels.

### Western blot analysis

Proteins were separated with 10% SDS-PAGE. Antibodies against MCT1, MCT2, MCT4, G6PAse (Santa Cruz, Heidelberg, Germany), PEPCK, AMPK and P-AMPK (Cell Signaling, Berverly, MA, USA) and β-Tubulin were used. After transfer and blocking, membranes were probed in 1% nonfat milk prepared in TBS-T with 1/1,000 rabbit anti-MCT1 or anti-MCT2^57^, or with 1/500 rabbit anti-MCT4 (Santa Cruz, Heidelberg, Germany), or with 1/1000 anti-AMPK or anti-pAMPK (Cell Signaling, Berverly, MA, USA) and with 1/10000 rabbit anti-β-Tubulin (Cell Signaling, Berverly, MA, USA) overnight at 4 °C. The specific band for each protein was detected using a goat anti-rabbit (1/10,000 in TBST-1X) peroxidase-conjugated secondary antibody (GE Healthcare, Piscataway, NJ, USA) incubated for 1 hour at room temperature. Bands were revealed with a chemiluminescence kit (BioRad, Reinach, Switzerland) and processed with a ChemiDoc XRS + system (BioRad, Reinach, Switzerland) for densitometry analysis.

### RNA extraction, reverse transcription and quantitative real time PCR

Brain or liver samples collected were lysed and homogenized in 350 μl of lysis buffer (RLT Buffer, Qiagen) using the Fast prep 24 lyzer (MPbio, Luzern, Switzerland) according to the manufacturer’s instructions. Total RNA was isolated on spin columns with silica-based membranes (RNeasy Mini Kit, Qiagen, Basel, Switzerland), following the manufacturer’s instructions. RNA was eluted with 30 μl of H_2_O. 200 ng of purified RNA was reverse transcribed in a volume of 50 μl using the RT High Capacity RNA-to-cDNA Kit (Applied Biosystems, Rotkreuz, Switzerland). Quantitative real-time PCR analysis was performed on cDNA obtained with the Applied Biosystems 7900 (Applied Biosystems, Rotkreuz, Switzerland) Real-Time PCR System using Power SYBR Green Taq polymerase master mix (Applied Biosystems, Rotkreuz, Switzerland). Primer sequences used for mRNA quantification were directed against NPY, AgRP, POMC, CART, BHBDH, HMGcs2, PEPCK, G6Pase, and Glycogen Phosphorylase mRNAs as well as *β*−2-microglobulin mRNA used as an endogenous control (See Table 1 for sequences). Data were analyzed with RQManager 1.2 software (Applied Biosystems, Rotkreuz, Switzerland) for relative quantitation of gene expression (RQ) using the 2^−ΔΔCT^ method^58^.

### Blood analysis

Insulinemia (ultra-sensitive Elisa Kit, Millipore, Zug, Switzerland), glucagonemia (Glucagon RIA, Millipore, Zug, Switzerland), lactatemia (The Edge analyser, Hasselt, Belgique), glycemia (Benecheck plus Multi-monitoring system, Hasselt, Belgique) and ketonemia (Free Style precision, Abbott, Baar, Switzerland) were measured after 24 hours of infusion using the indicated kits.

### Liver glycogen measurement

Briefly, 100 mg of tissue stored at −80 °C were homogenized in citrate buffer (NaF 50 mM, citric acid 100 mM, pH 4.2) and then centrifuged at 5000 g for 10 minutes at 4 °C. The supernatant was removed, and 460 μl of it were incubated with 40 μl of a solution of amyloglucosidase 50 U/ml (Sigma, Buchs, Switzerland) diluted in sodium citrate buffer, while another 460 μl were incubated with 40 μl of sodium citrate buffer only. The tubes were shaken for 30 minutes at 55 °C. Then, 10 μl of each sample were deposited in 96-well plates with 200 μl of a Ready to Use buffer (BioMerieux, Geneva, Switzerland), incubated at room temperature for 20 minutes. The optical density was read at 505 nm by spectrophotometry. The difference between the condition with amyloglucosidase and the condition with buffer only represents the glycogen content of the liver sample. Glycogen content is expressed as milligrams of glucose resulting from glycogen hydrolysis per gram of tissue.

### Statistical analysis

Results are presented as mean ± SEM. Statistical analysis was performed using Prism 6.01. Normality has been tested with a Kolmogorov-Smirnov test. An unpaired Student t-test was used for all the experiments except for the tolerance tests for which a two-way ANOVA was used. Significant differences are indicated as *, **, or *** on graphic representations for p values < 0.05, 0.01, or 0.001, respectively.

## Additional Information

**How to cite this article**: Carneiro, L. *et al*. Hypothalamic sensing of ketone bodies after prolonged cerebral exposure leads to metabolic control dysregulation. *Sci. Rep.* **6**, 34909; doi: 10.1038/srep34909 (2016).

## Figures and Tables

**Table 1:** Primer sequences for the various gene mRNAs analyzed in the study.

Name	Forward sequence	Reverse sequence
NPY	ATGCTAGGTAACAAGCGAATGG	TGTCGCAGAGCGGAGTAGTAT
Agrp	ATGCTGACTGCAATGTTGCTG	CAGACTTAGACCTGGGAACTCC
CART	CCCGAGCCCTGGACATCTA	GCTTCGATCTGCAACATAGCG
POMC	ATGCCGAGATTCTGCTACAGT	TCCAGCGAGAGGTCGAGTTT
BHBDH	TGCAACAGTGAAGAGGTGGAGAAG	CAAACGTTGAGATGCCTGCGTTGT
HMGcs2	TGGTTCAAGACAGGGACACAGAAC	AGAGGAATACCAGGGCCCAACAAT
PEPCK	GGCCCCGGGAGTCACCATCA	TGCCGAAGTTGTAGCCGAAGAAGG
G6Pase	AACGTCTGTCTGTCCCGGATCTAC	ACCTCTGGAGGCTGGCATTG
Glycogen Phosphorylase	GCCAGCGCCTCGGGGTAATC	GCCACGCGGTGAACGGTGTA
β-2 Microglobulin	CCCCACTGAGACTGATACATACG	CGATCCCAGTAGACGGTCTTG
